# Binarized neural network of diode array with high concordance to vector–matrix multiplication

**DOI:** 10.1038/s41598-024-56575-4

**Published:** 2024-03-11

**Authors:** Yunwoo Shin, Kyoungah Cho, Sangsig Kim

**Affiliations:** Department of Electrical Engineering, Korea University, 145 Anam-ro, Seongbuk-gu, Seoul, 02841 Republic of Korea

**Keywords:** Binarized neural network, Multiply-accumulate, Vector–matrix multiplication, Gated p^+^-n-p-n^+^ diodes, Positive-feedback loop mechanism, Applied physics, Electronics, photonics and device physics, Electrical and electronic engineering

## Abstract

In this study, a binarized neural network (BNN) of silicon diode arrays achieved vector–matrix multiplication (VMM) between the binarized weights and inputs in these arrays. The diodes that operate in a positive-feedback loop in their p^+^-n-p-n^+^ device structure possess steep switching and bistable characteristics with an extremely low subthreshold swing (below 1 mV) and a high current ratio (approximately 10^8^). Moreover, the arrays show a self-rectifying functionality and an outstanding linearity by an R-squared value of 0.99986, which allows to compose a synaptic cell with a single diode. A 2 × 2 diode array can perform matrix multiply-accumulate operations for various binarized weight matrix cases with some input vectors, which is in high concordance with the VMM, owing to the high reliability and uniformity of the diodes. Moreover, the disturbance-free, nondestructive readout, and semi-permanent holding characteristics of the diode arrays support the feasibility of implementing the BNN.

## Introduction

With the explosive growth of data, brain-inspired (i.e. neuromorphic) computing systems have achieved significant improvements in parallel computing and efficient data processing^1–5^. In particular, binarized neural networks (BNNs) have recently demonstrated their capabilities in image recognition applications^6–10^. The accelerators in the BNNs perform a matrix "multiply accumulate" (MAC) operation (i.e. vector–matrix multiplication (VMM)) between the binarized weights and analog inputs^6,7^. The bitwise MAC operation enables extensive applicability to resource-constrained platforms, such as edge devices and mobile processors, promising a considerable reduction in memory (approximately 32 ×) and computation (approximately 2 ×) requirements compared with other neural networks^6–10^. Furthermore, it is still difficult for emerging synaptic devices to fully implement analog neural networks (analog input and analog weight) with nonlinear conductance changes and device variations^1–4,11–13^. However, digital synaptic devices are suitable for implementing BNNs because of their binarized weights^13–16^.

Various memory devices, such as resistive random-access memories (RRAMs), magnetoresistive RAMs (MRAM), flash-based memory devices, static RAMs (SRAM), and dynamic RAMs (DRAMs), have been widely researched for BNN implementation^11–21^. Their definite bistable characteristics, with a high on/off ratio and input/output linearity, are required for the implementation^13–15^. However, despite their improved characteristics, these devices still do not satisfactorily meet this requirement^13–17^. Moreover, as the number of hidden layers in the multilayer perceptron structure of neural networks increases, the neuron circuits that interconnect the pre- and post-layers become more complicated, which degrades the area and power efficiencies of computing systems based on neural networks^21–24^. The activation function of neuronal circuits is simplified or merged into a memory array to alleviate degradation^25–27^.

Recently, silicon diodes operating in a positive-feedback loop mechanism have demonstrated their feasibility as neuromorphic devices, owing to their superior electrical characteristics, high reliability, and reproducibility^28–32^. The positive-feedback loop mechanism enables diodes to exhibit steep switching and bistable characteristics with a high ON/OFF ratio^32^. Compared to other memory devices used as components of emerging BNNs^11–21^, the p^+^-n-p-n^+^ diodes operating by the positive-feedback loop mechanisms have a significant advantage in terms of reliability and endurance since the relatively low operating voltage prevents the deterioration of devices under sustained operations. Furthermore, the BNN comprising the p^+^-n-p-n^+^ diodes can be more simplified than those designed by SRAMs and DRAMs^18,33^. In this study, we demonstrate BNNs with 4 × 1 and 2 × 2 arrays, consisting of p^+^-n-p-n^+^ diodes.

## Results

**Figure 1:**
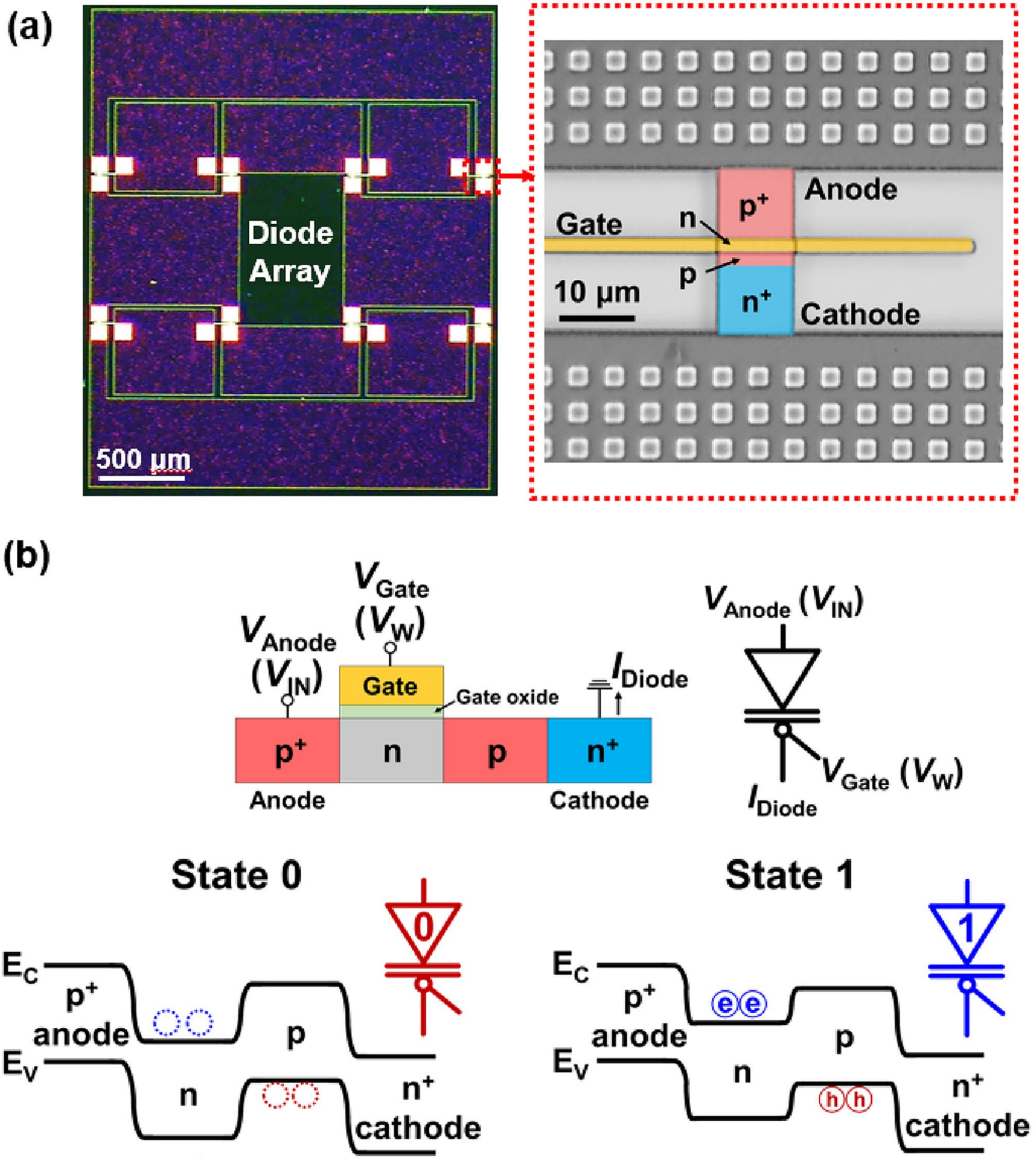
Optical images of (**a**) diode array with p^+^-n-p-n^+^ diode, and (**b**) diode schematic, energy-band diagrams in States 0 and 1, and red (blue) circuit symbol of State 0 (State 1). The n-doped region in the optical image is present beneath the yellow-colored gate electrode.

Optical images of the diode array and the p^+^-n-p-n^+^ diode in this array are shown in Fig. 1a. The bistable characteristic principle of the diode is illustrated in Fig. 1b with a diode schematic, energy-band diagrams in State 0 and State 1, and the red (blue) circuit symbol of State 0 (State 1). For the diode in the array, the gate voltage (*V*_Gate_) controls the injection and accumulation of charge carriers by modulating the potential barrier in the n-doped region of the energy-band diagram. In State 0, excess charge carriers are absent in the potential wells in the n- and p-doped regions of the energy-band diagram, preventing the diode current (*I*_Diode_) from flowing in the diode. In contrast, in State 1, excess charge carriers accumulate in the potential wells in the n- and p-doped regions in the energy-band diagram, resulting in the *I*_Diode_ to flow in the diode. Modulation of the potential barrier allowed the diode to exhibit bistable characteristics. However, for the BNN operation, the conductance in State 0 (State 1) corresponds to Weight 0 (1), and the anode voltage (*V*_Anode_) and *V*_Gate_ represent the input and weight voltages (*V*_IN_ and *V*_W_), respectively.

**Figure 2:**
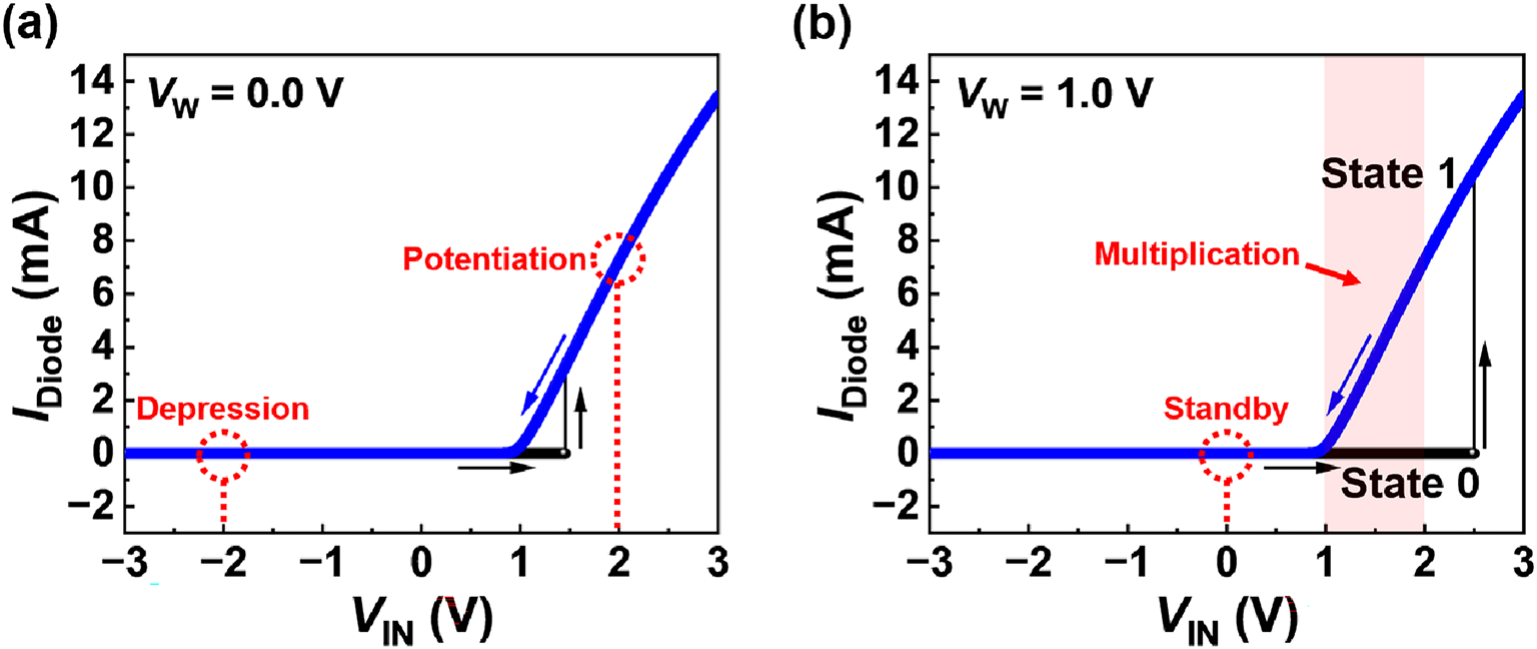
*I*_Diode_ vs. *V*_IN_ curves for a diode at (**a**) *V*_W_ = 0.0 V and (**b**) *V*_W_ = 1.0 V. The black and blue curves indicate the forward and reverse sweeps of *V*_IN_, respectively. The BNN operating conditions are shown in the figures.

Figure 2 shows the *I*_Diode_ versus *V*_IN_ curves for a diode at *V*_W_ = 0.0 V and *V*_W_ = 1.0 V. The BNN operating conditions are illustrated in the figure. As *V*_IN_ increases from − 3 to 3 V (black curves), *I*_Diode_ first remains at 0 mA, after which it abruptly increases at a *V*_IN_ of 1.5 V for *V*_W_ = 0.0 V and a *V*_IN_ of 2.5 V for *V*_W_ = 1.0 V. The generation of the positive-feedback loop leads to an abrupt increase in the *I*_Diode_. As *V*_IN_ decreased from 3 to − 3 V (blue curves), *I*_Diode_ linearly decreased and reached 0 mA near *V*_IN_ = 1 V; the positive-feedback loop was eliminated near *V*_IN_ = 1 V. The bistable characteristics are presented in the *I*_Diode_ versus *V*_IN_ curves, and the high ratio of the current magnitudes of States 1 and 0 is approximately 10^8^ at a *V*_W_ of 1.0 V for *V*_IN_ = 2.0 V (see Supporting Information, Fig. S1). The unipolar switching (i.e. rectifying) characteristics inherit the electrical properties of the p–n diode. Regardless of *V*_W_, the *I*_Diode_ versus *V*_IN_ curve shape in State 1 resembles that of the rectified linear unit function used for the activation function of neural networks, including BNNs^34–36^.

In Fig. 2a, the diode state becomes State 1 (State 0) by the potentiation (depression) at a *V*_IN_ of 2.0 V (− 2.0 V), and *V*_W_ of 0.0 V. The potentiated (depressed) diode weighed 1 (0). In Fig. 2b, the potentiated or depressed diode is in a standby state at *V*_W_ = 1.0 V and *V*_IN_ = 0.0 V. For a *V*_IN_ range of 1.1 V to 2.0 V, the *I*_Diode_ of the potentiated diode is linearly proportional to *V*_IN_, the *I*_Diode_ of the depressed diode remains at a low level, and the difference in the *I*_Diode_ of the potentiated and depressed diodes is used for the MAC operation.

**Figure 3:**
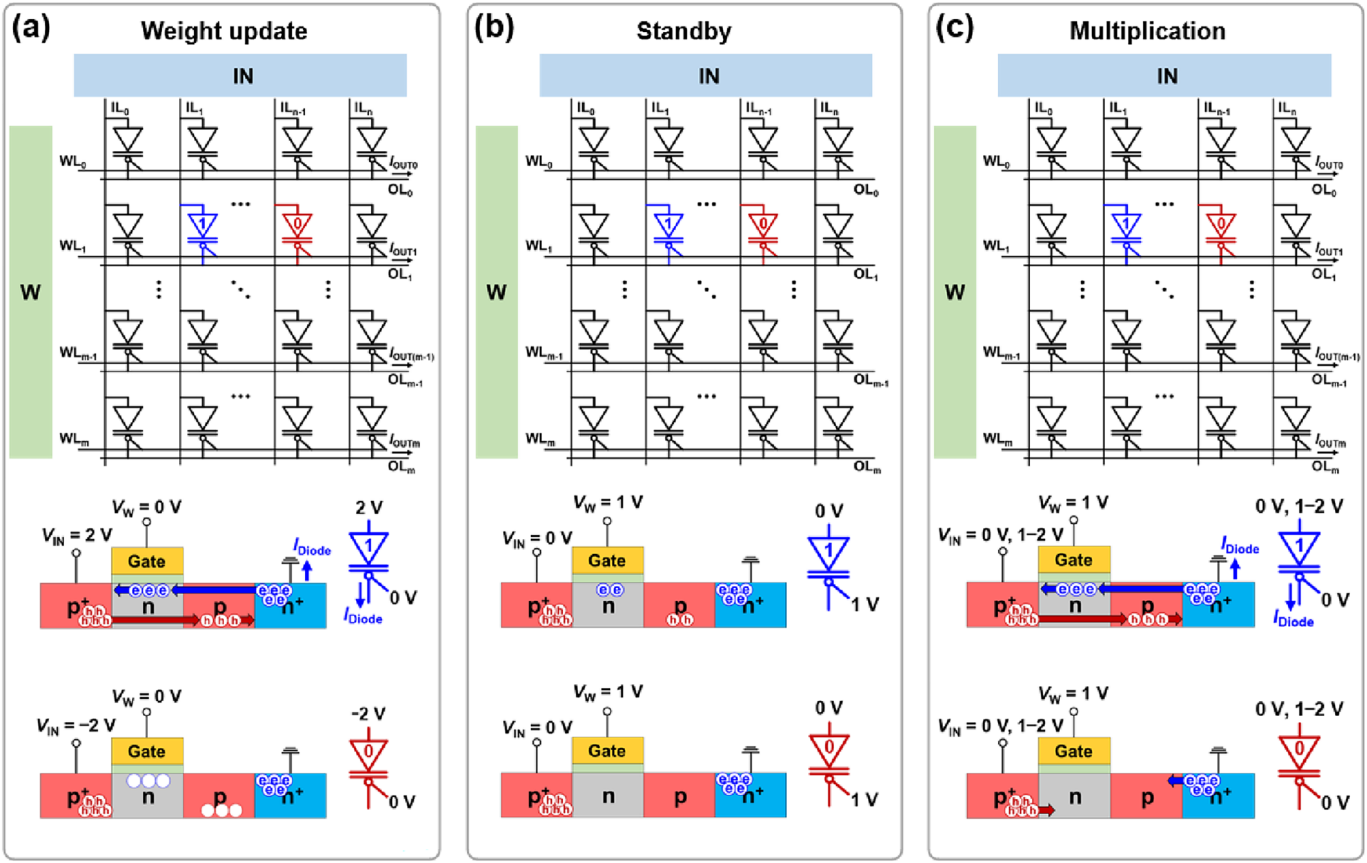
Diode-array architecture, schematics, and circuit symbols of diodes in (**a**) weight update, (**b**) standby, and (**c**) multiplication operations. Colored boxes with W and IN mean the peripheral parts of the weight and input lines (WLs and ILs), respectively.

To describe the BNN operation, the diode-array architecture, schematics, and circuit symbols of the p^+^-n-p-n^+^ diodes in the weight update, standby, and multiplication operations are shown in Fig. 3a–c. In the diode array, p^+^, n^+^, and gate electrodes were connected to the input lines (ILs), output lines (OLs), and weight lines (WLs), respectively. WLs and OLs were parallel to each other and perpendicular to ILs^37–39^. The multiplication of *V*_IN_ and the conductance (i.e. weight) of a diode using Ohm’s law produced *I*_Diode_, and the summation of *I*_Diode_ at each common OL (i.e. the same row in the diode array) using Kirchhoff’s law yielded the output current (*I*_OUT_). Thus, the diode-array architecture can perform parallel MAC operations. For the BNN operations, the* V*_IN_ for IL, *V*_W_ for WL, and *I*_OUT_ for OL signals represent the input, weight update selection, and output, respectively.

In the weight update operation in Fig. 3a, the binarized weight (**W**) matrix is updated by both *V*_IN_ (2.0 V or − 2.0 V) and *V*_W_ (0.0 V). The diodes selected in the array with *V*_W_ = 0.0 V were potentiated (depressed) at *V*_IN_ of 2.0 V (− 2.0 V). In the standby operation shown in Fig. 3b, all *V*_W_ and *V*_IN_ are 1.0 V and 0.0 V, respectively. The diodes maintained their memory states with extremely low *I*_Diode_. In the multiplication operation shown in Fig. 3c, the *V*_W_ (1.0 V) and *V*_IN_ (0.0 V or from 1.1 V to 2.0 V) are applied to the diode array. *I*_Diode_ represents the output of the product of *V*_IN_ and the binarized weight, and *I*_OUT_ is obtained from the summation of the *I*_Diode_.

**Figure 4:**
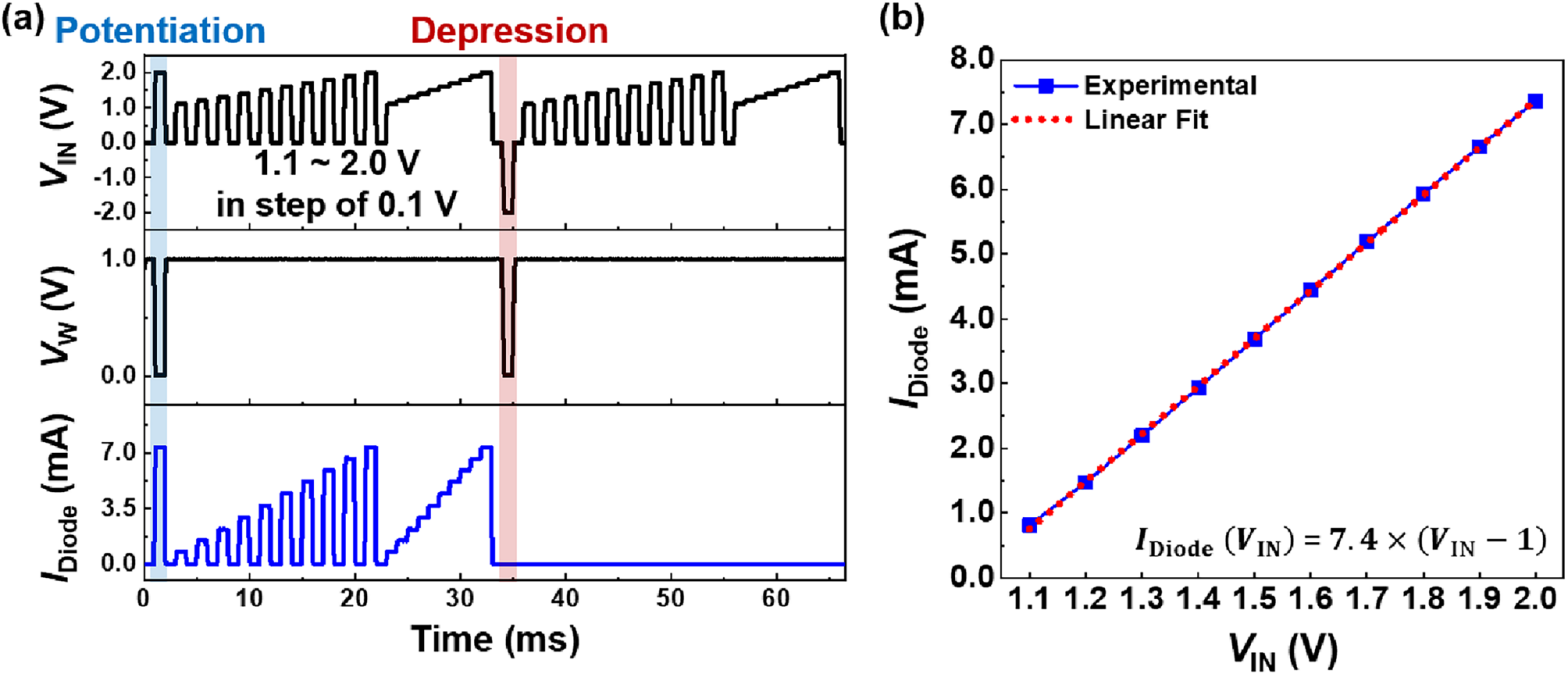
BNN operation of a diode. (**a**) *I*_Diode_ for potentiated and depressed diodes and (**b**) *I*_Diode_ vs. *V*_IN_ diode characteristics of potentiated diode. Here, the R-square value of the linear fit is 0.99986, which means that the diode features high linearity.

Figures 4 shows the BNN operation and *I*_Diode_ versus *V*_IN_ characteristics of the selected diode in an array. In Fig. 4a, for potentiation (depression), voltage pulses with *V*_IN_ = 2.0 V (− 2.0 V) and *V*_W_ = 0.0 V were applied to the diode. After the potentiation, the *I*_Diode_ is linearly proportional to the multipulse and staircase waveforms of *V*_IN_ ranging from 1.1 to 2.0 V in steps of 0.1 V. In contrast, after the depression, the *I*_Diode_ remained at a low level despite the same *V*_IN_ pulses as the potentiation. During the standby operation between the* V*_IN_ pulses, the diode memorized the weight with a power consumption of approximately 0 W. In Fig. 4b, the extracted *I*_Diode_ values of the potentiated diode for each *V*_IN_ pulse can be expressed as a linear function of *V*_IN_, as follows:

1 $${I}_{{\text{Diode}}} \left({V}_{{\text{IN}}}\right)=7.4\times \left({V}_{{\text{IN}}} -1\right).$$

Here, the coefficient 7.4 represents the conductance of the potentiated diode (i.e. Weight 1). For the depressed diode (i.e. Weight 0), the conductance is close to 0, and the *I*_Diode_ remains at a low level during multiplication after depression. The constant term – 1 indicates the lower limit of the *V*_IN_ range for diode multiplication. This expression implies that the single diode performs multiplication between *V*_IN_ and weight with outstanding linearity.

**Figure 5:**
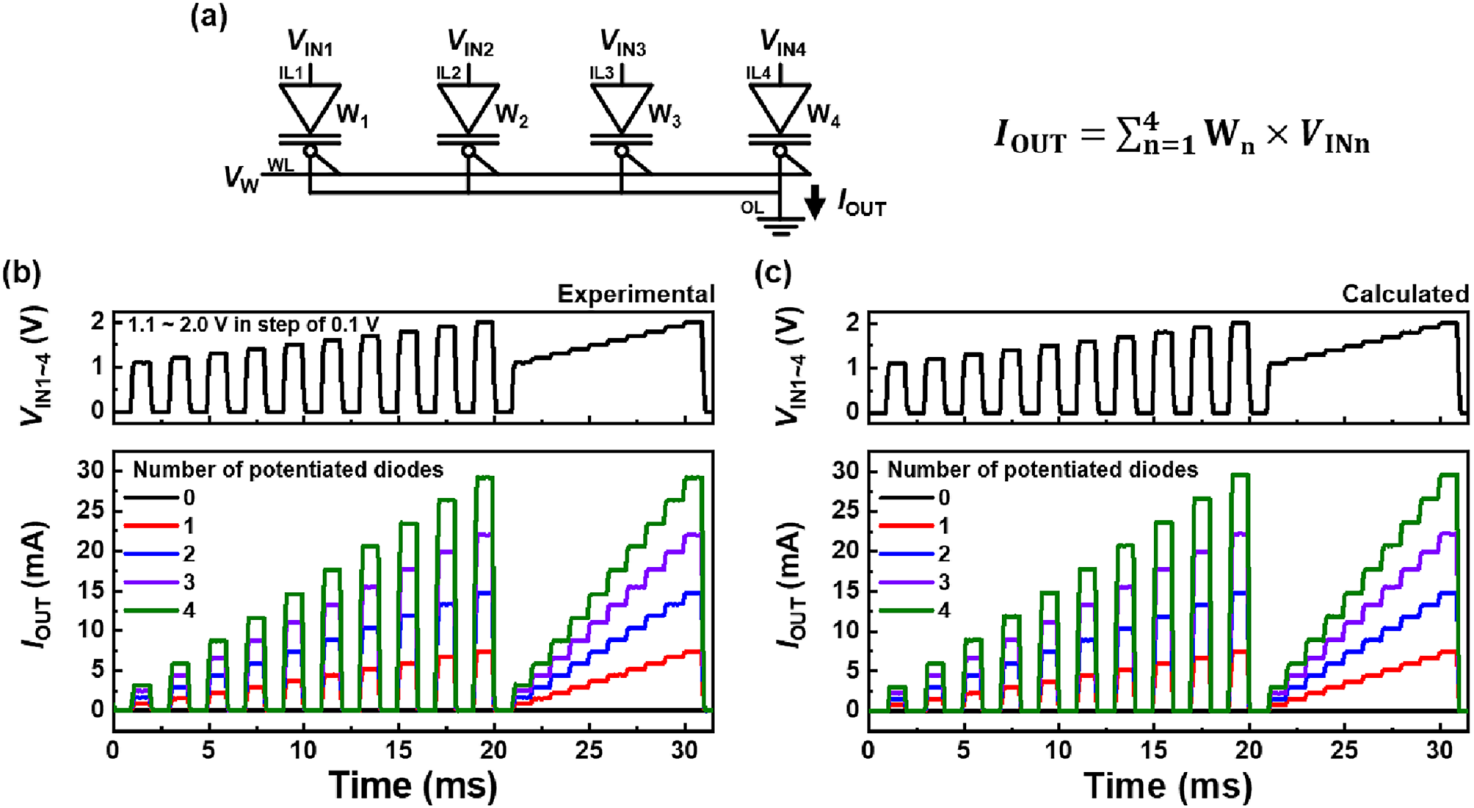
BNN operation of 4 × 1 diode array. (**a**) Circuit diagram of array, (**b**) experimental data of bitwise MAC operation, and (**c**) calculated data of bitwise MAC operation. Here, all *V*_IN_ (*V*_IN1_–*V*_IN4_) are identical.

The bitwise MAC operation of the BNN in a 4 × 1 diode array is illustrated in Fig. 5. The MAC operation was conducted by multiplying the input *V*_INn_ and binarized weight W_n_ for each n^th^ diode. In a 4 × 1 diode array in Fig. 5a, *I*_OUT_ can be expressed as

2 $${I}_{{\text{OUT}}}=\sum_{n=1}^{4}{{\text{W}}}_{{\text{n}}}\times {V}_{{\text{INn}}}.$$

The experimental data for the MAC operation are shown in Fig. 5b. All *V*_IN_ were applied in the same waveforms as those in Fig. 4a. W_n_ is updated before the MAC operation, and *I*_OUT_ remains linearly proportional to the multipulse and staircase waveforms of *V*_IN_. Moreover, *I*_OUT_ is linearly proportional to the number of potentiated diodes for the same *V*_IN_ pulse, and there are few electrical variations between the component diodes in the array, owing to the wafer-scale full CMOS process. Here, the product of *V*_Inn_ and W_n_ can be replaced with the *I*_Diode_ in Eq. (1). Thus, Eq. (2) becomes

3 $${I}_{{\text{OUT}}}={I}_{{\text{Diode}}}\times {\text{n}}, \mathrm{where \,\,n}=\#\mathrm{ of \,\,potentiated \,\,diodes}.$$

The MAC operation data (obtained from Eq. (3)) plotted in Fig. 5c were in good agreement with the experimental data in Fig. 5b. The uniformity of the component diodes in the array can minimize the BNN computational errors^39–41,42^. Thus, a massive array architecture comprising gated p^+^-n-p-n^+^ diodes can be realized with a high ratio of the bistable current magnitude to uniformity.

**Figure 6:**
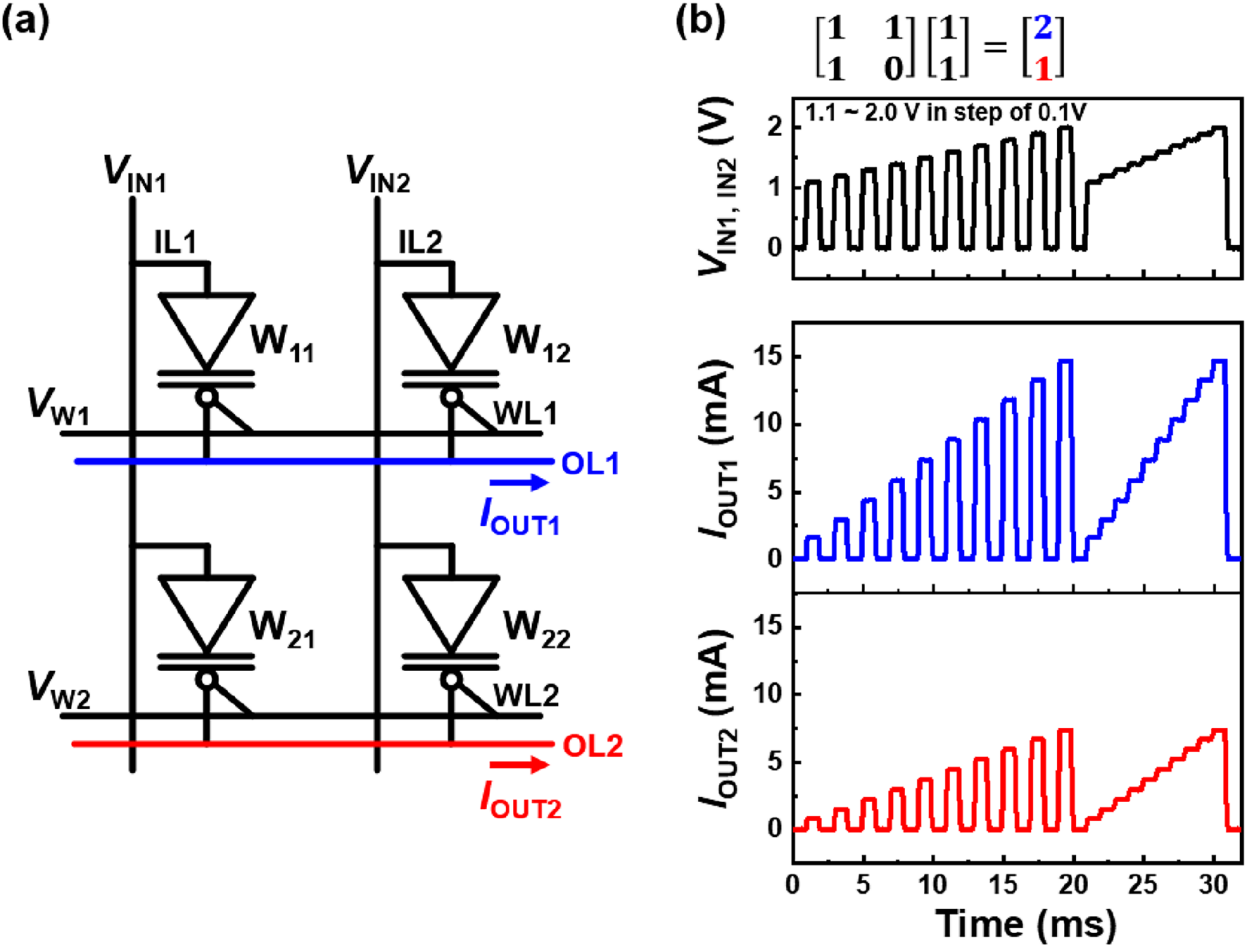
BNN operation of 2 × 2 diode array. (**a**) Circuit diagram of array and (**b**) matrix MAC operation with fixed **W** matrix and **IN** vector. Here, *V*_IN1_ and *V*_IN2_ are identical.

A 2 × 2 diode array is used to perform the matrix MAC operation of the BNN, as shown in Fig. 6. The binarized weights of the diodes in Fig. 6a are the components of the **W** matrix $$\left[\begin{array}{cc}{{\text{W}}}_{11}& {{\text{W}}}_{12}\\ {{\text{W}}}_{21}& {{\text{W}}}_{22}\end{array}\right]$$, where *I*_OUT1_ (${I}_{{\text{Diode}}}\times {\text{n}}={{\text{W}}}_{11}\times {V}_{{\text{IN}}1}+{{\text{W}}}_{12}\times {V}_{{\text{IN}}2}$) and *I*_OUT2_ (${I}_{{\text{Diode}}}\times {\text{m}}={{\text{W}}}_{21}\times {V}_{{\text{IN}}1}+{{\text{W}}}_{22}\times {V}_{{\text{IN}}2}$) are obtained via the OLs, and n(m) is the number of potentiated diodes in the first (second) row in the array. In this array, the IN-vector component IN_1_ or IN_2_ is 0 (if *V*_IN_ is 0.0 V) or 1 (if *V*_IN_ is from 1.1 V to 2.0 V), and the OUT-vector component OUT_1_ (OUT_2_) is n (m). Thus, the matrix MAC operation can be expressed as $$\left[\begin{array}{cc}{{\text{W}}}_{11}& {{\text{W}}}_{12}\\ {{\text{W}}}_{21}& {{\text{W}}}_{22}\end{array}\right]\left[\begin{array}{c}{{\text{IN}}}_{1}\\ {{\text{IN}}}_{2}\end{array}\right]= \left[\begin{array}{c}{{\text{OUT}}}_{1}\\ {{\text{OUT}}}_{2}\end{array}\right]$$, which represents the VMM between the weight matrix and input vector, **W **$\cdot$** IN**^**T**^** = OUT**^**T**^.

**Figure 7:**
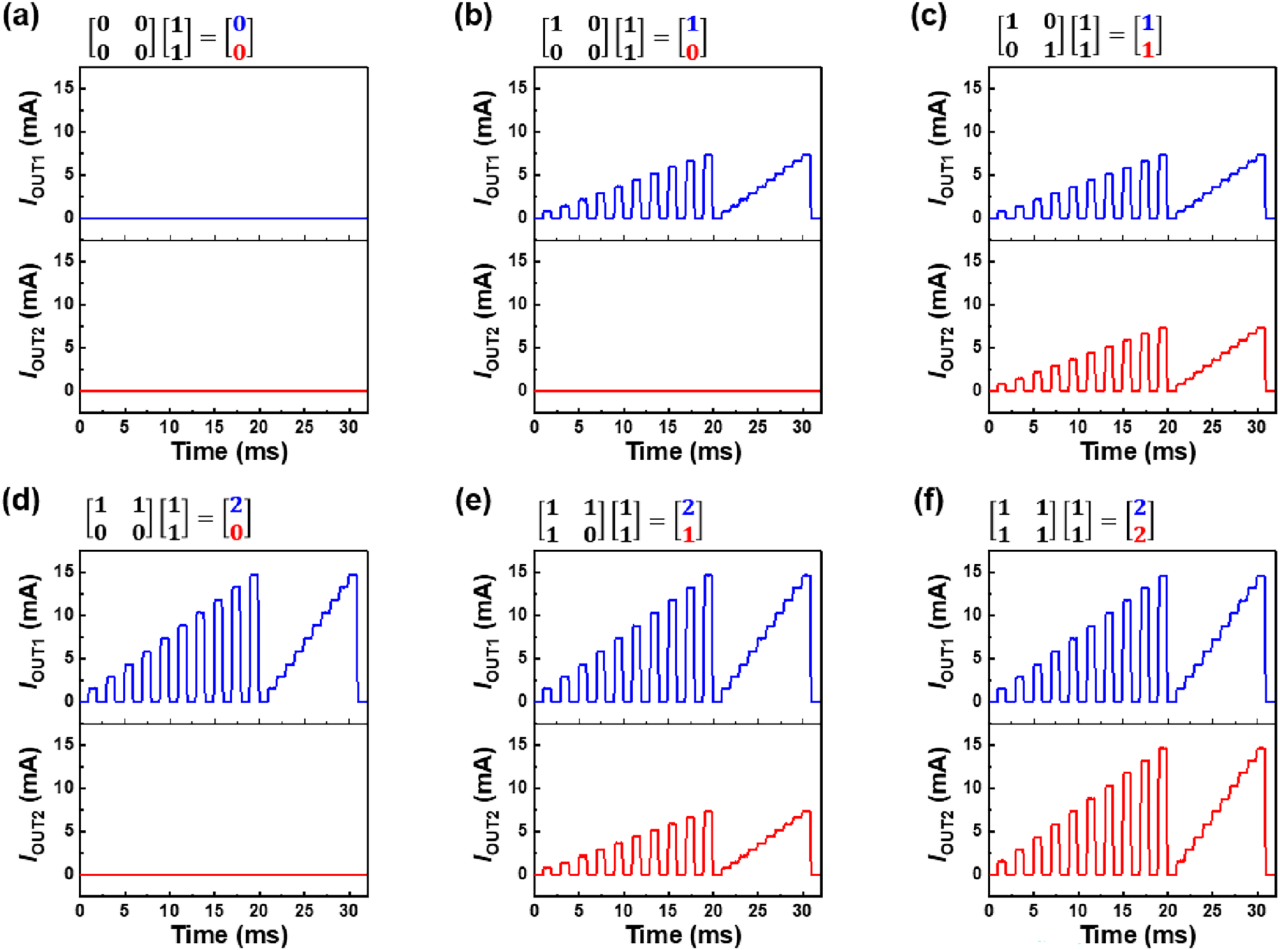
Matrix MAC operations of 2 × 2 diode array for (**a**–**f**) six different **W** cases with fixed **IN** vector. The other cases are excluded because their results of the matrix MAC operations are the same as these six cases.

The voltage pulses of *V*_IN_ (2.0 V or − 2.0 V) and *V*_W_ (0 V) are applied to the diode array to update the **W** matrix, as shown in Fig. 6a. In Fig. 6b, the 2 × 2 diode array performs the matrix MAC operation in the **W** = $$\left[\begin{array}{cc}1& 1\\ 1& 0\end{array}\right]$$ case among the 16 different **W** cases (see Fig. 7 for other **W** cases). **W** is updated to the **W** = $$\left[\begin{array}{cc}1& 1\\ 1& 0\end{array}\right]$$ case, after which *V*_IN1_ and *V*_IN2_ are applied to the same waveforms depicted in Fig. 4a for the matrix MAC operations. Thus, *I*_OUT1_ was twice as large as *I*_OUT2_. Consequently, the diode array exhibits a simplified VMM of $$\left[\begin{array}{cc}1& 1\\ 1& 0\end{array}\right]\left[\begin{array}{c}1\\ 1\end{array}\right]= \left[\begin{array}{c}2\\ 1\end{array}\right]$$.

In Fig. 7, we demonstrate the matrix MAC operations of the 2 × 2 diode array for six different **W** values. The IN-vector components IN_1_ and IN_2_ are both 1, where *I*_OUT1_ and *I*_OUT2_ correspond to the outputs OUT_1_ and OUT_2_ of the matrix MAC operation for each **W** case, respectively. Here, *V*_IN1_ and *V*_IN2_ are applied to the same waveforms as those in Fig. 4a. *I*_OUT1_ and *I*_OUT2_ in Fig. 7e are identical to those in Fig. 6c. For each **W** case, the output of the matrix MAC operation has a high concordance with the simplified VMM equation in each figure owing to the high uniformity of the diodes.

**Figure 8:**
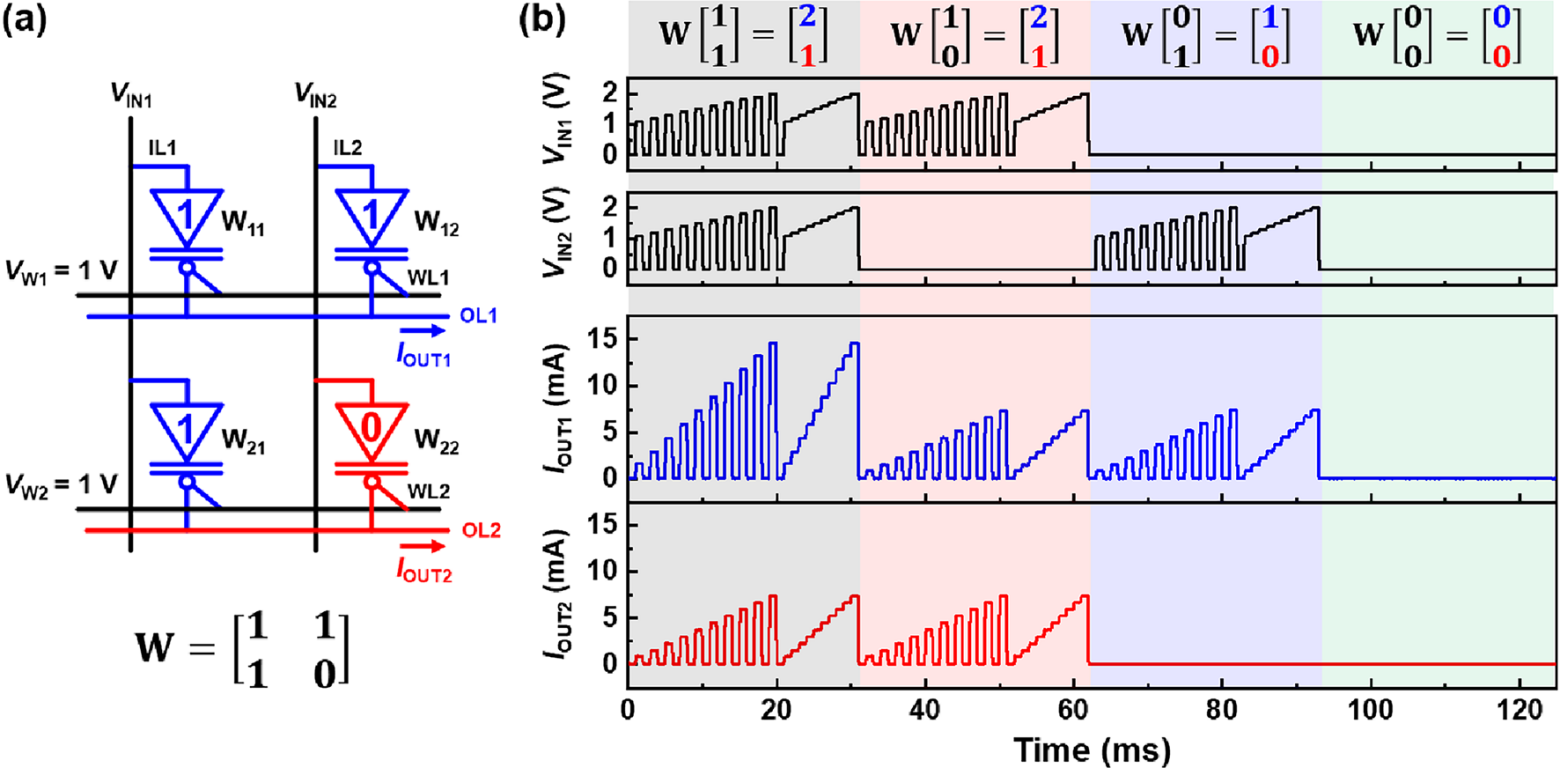
Matrix MAC operations of 2 × 2 diode array with fixed **W** matrix. (**a**) Circuit diagram of array and (**b**) matrix MAC operations for four different **IN** vectors. The **W** matrix is updated before the matrix MAC operations.

For the **W** matrix $$\left[\begin{array}{cc}1& 1\\ 1& 0\end{array}\right]$$ in Fig. 8a, the OUT-vector components OUT_1_ and OUT_2_ vary with the IN-vector components IN_1_ and IN_2_, which are highly concordant with the simplified VMMs in Fig. 8b. *I*_OUT1_ and *I*_OUT2_ depend on the number of potentiated diodes in the array and the multipulse and staircase waveforms of *V*_IN1_ and *V*_IN2_. After multiplication, the diode array maintains the binarized weight matrix owing to the nondestructive readout characteristics of the component diodes^43^. Instead, the component diodes refresh their weights during multiplication operations. They also exhibit superior electrical stability against bias stresses (continuous input pulses), unlike other memory devices^13,19–21,44,45^. The diode array holds the binarized weight matrix semi-permanently using refresh operations while performing multiplication operations. Moreover, the binarized weights are not updated unless *V*_IN_ and *V*_W_ are applied simultaneously. The binarized weight matrix did not change for multiplication. However, for the weight update operations, the matrix is determined by *V*_W1_ and *V*_W2_.

## Discussion

We demonstrated the BNN operation of an array composed of p^+^-n-p-n^+^ diodes with bistable characteristics. The component diode exhibited inherent unipolar switching characteristics, and the array was immune to sneak path problems, unlike previously proposed BNNs^11–13,16,43^. In addition to the outstanding bistable characteristics with a high current ratio (approximately 10^8^), the rectifying characteristics can simplify peripheral neuron circuits. Moreover, the simplified peripheral neuron circuits help to reduce the burden of area and power consumption for BNN-based computing systems. The diode array performed matrix MAC operations with high concordance with the VMM between the binarized weight matrix and input vector. Furthermore, the diode array exhibited nondestructive readout and semi-permanent holding characteristics. Our diodes can realize the compact synaptic array in which the cell is composed of a single device with 6F^2^ (F = feature size) as well as operate at relatively low voltages (≤ 2 V). Consequently, p^+^-n-p-n^+^ diodes are the most suitable building blocks for area-/energy-efficient and reliable synaptic arrays in BNN.

## Methods

### Device fabrication

The p^+^-n-p-n^+^ diodes were fabricated using full CMOS processes from a p-type (100)-oriented silicon-on-insulator wafer with a 340-nm thick top Si layer on a 2-μm thick buried oxide layer. First, the wafer was cleaned by standard clean-1 and 50:1 diluted HF to remove particles or impurities. A 10-nm thick SiO_2_ layer was grown on the Si surface as a sacrificial layer to minimize channeling and damage to the channel during ion implantation by dry oxidation (800 °C, 200 min). The n-doped regions were formed using a conventional ion implantation process, in which P^+^ ions were implanted at a dose of 3 × 10^13^ cm^−2^ with an ion energy of 60 keV. Subsequently, the wafer was annealed at 1100 °C for 30 min under ambient nitrogen. The 25-nm thick SiO_2_ layers were thermally grown as the gate dielectric for the gated p^+^-n-p-n^+^ diodes by dry oxidation (850 °C, 270 min). A 400-nm thick and 1.5-μm wide poly-Si gate electrode was formed by low-pressure chemical vapor deposition (LPCVD), followed by photolithography, and a dry etching process was used to delineate the gate profile. The lightly doped drain extension regions were implanted with ${\text{BF}}_{2}^{+}$ ions at a dose of 1 × 10^12^ cm^−2^ and an ion energy of 10 keV. And then LPCVD-based tetraethyl orthosilicate (TEOS) was used to form the 200-nm thick gate sidewall spacers. For the p-doped regions, ${\text{BF}}_{2}^{+}$ ions at a dose of 3 × 10^13^ cm^−2^ were implanted at an ion energy of 40 keV. The n^+^ cathode, heavily n-doped poly-Si gate, and p^+^ anode regions were formed by masked ion implantation (P^+^ at 3 × 10^15^ cm^−2^, 50 keV and ${\text{BF}}_{2}^{+}$ at 3 × 10^15^ cm^−2^, 30 keV). Thereafter, the wafer was annealed at 1000 °C for 30 min in ambient nitrogen, followed by rapid thermal annealing at 1050 °C for 30 s to activate the implanted dopants with uniform diffusion and eliminate defects. The dual-step annealing process was carried out to form the p^+^-n-p-n^+^ doping structure. Finally, a Ti/TiN/Al/TiN metal alloy was deposited in the drain, source, and gate contact regions after deposition of a 700-nm thick interlayer dielectric layer (for device protection) using the LPCVD-based TEOS.

### Measurement

All electrical data were acquired at room temperature using a semiconductor parameter analyzer (HP4155C, Agilent Technologies) and Keithley 2636A and 2636 B source meters. The input-pulse width for the diode and MAC array operations was selected as 1 ms because of the limitations of the Keithley source meters. The experimental set-up to examine the BNN operations of the diode array is shown in Fig. S2 of supporting information.

### Supplementary Information

Supplementary Information.

## Data Availability

All data generated during this study are included in this published article (and its Supporting Information files).
