# Supplementary material for: Binarized neural network of diode array with high concordance to vector–matrix multiplication
Source: Sci Rep. 2024 Mar 11;14:5891. doi: 10.1038/s41598-024-56575-4 (PMC10928169; doi:10.1038/s41598-024-56575-4)
Supplement: Supplementary file 1 — Supplementary Information. [file 41598_2024_56575_MOESM1_ESM.pdf]

Supporting Information

Binarized Neural Network of Diode Array with High Concordance to Vector-Matrix Multiplication

Yunwoo Shin, Kyoungah Cho, and Sangsig Kim*

Department of Electrical Engineering, Korea University 145 Anam-ro, Seongbuk-gu, Seoul 02841, Republic of Korea.

*Corresponding author. Tel: +82-2-3290-3245; Fax: +82-2-3290-3894

E-mail address: sangsig@korea.ac.kr

S1. I_{Diode} versus V_{IN} characteristics for p$^+$-n-p-n$^+$ diode

The memory state of a p$^+$-n-p-n$^+$ diode can be updated by modulating the height of the potential barrier in the n-doped region using a V_W sweep, as shown in Fig. S1. As the V_W is swept from 1.5 V to −1.5 V for V_{IN} = 2.0, the potential barrier in the n-doped region decreases. The I_{Diode} abruptly increases with an extremely low subthreshold swing below approximately 1 mV/dec at V_W = 0.5 V, owing to the generation of a feedback loop in the p$^+$-n-p-n$^+$ silicon layer of the diode. The bistable characteristics of the diode were observed with a high ratio (approximately 10^8) of the current magnitudes of States 1 and 0 at V_W = 1.0 V.

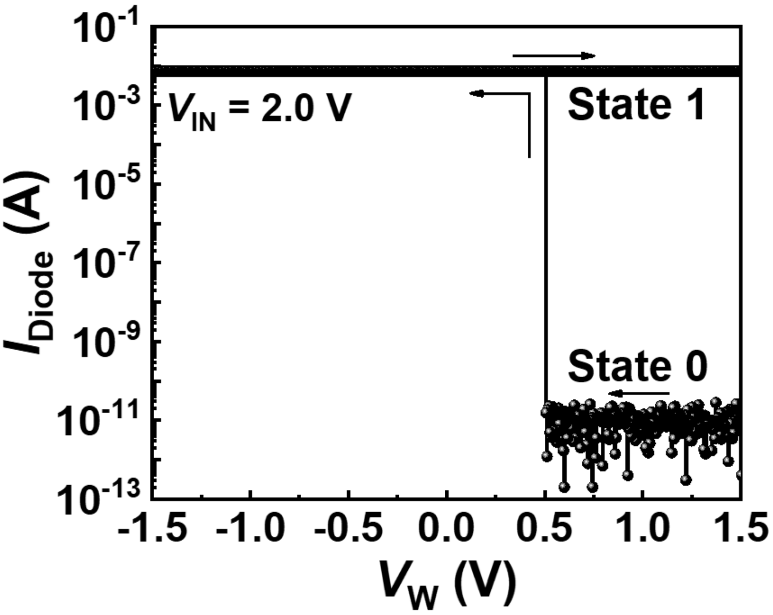

Figure S1. I_{Diode} vs. V_W characteristics of p$^+$-n-p-n$^+$ diode for V_{IN} = 2.0 V

S2. Experimental set-up for the BNN operations of the diode array

Figure S2 shows the experimental set-up to examine the BNN operations (weight update, standby, and multiplication) of the diode array. On the probe station, a probe card was used to contact the electrodes (ILs, WLs, and SLs) of the array fabricated on the 6-inch wafer. The voltages (V_{IN} and V_W) and currents (I_{OUT}) were applied and measured, respectively, by Keithley 2636A and 2636B source meters through the 8 cables. Using a measurement software, the source meters were set to volt- or current-meter modes, and measured data were stored as excel files.

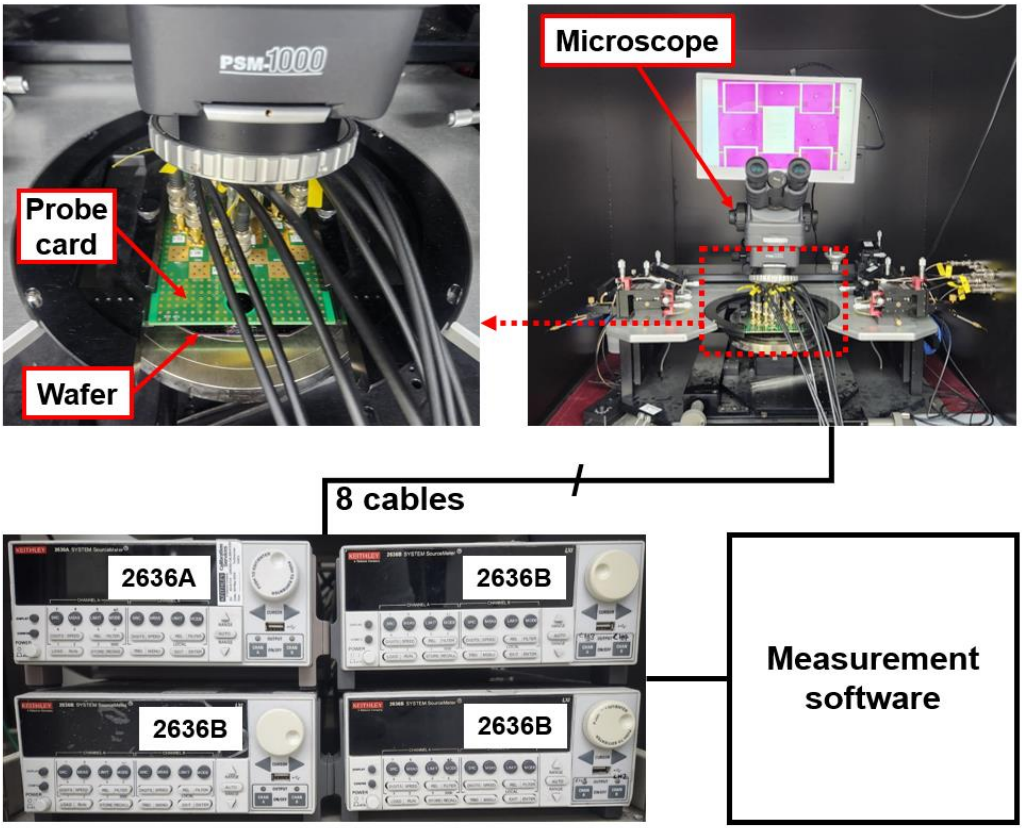

Figure S2. Experimental set-up for the BNN operations of the diode array
